# Job Characteristics, Well-Being and Physical Activity: A Field Study Using a Consumer Fitness Tracker

**DOI:** 10.5964/ejop.2447

**Published:** 2021-11-30

**Authors:** Nina Raffaela Grossi, Fabiola Gattringer, Bernad Batinic

**Affiliations:** 1 Department of Work, Organizational and Media Psychology, Johannes Kepler University, Linz, Austria; University of Wollongong, Wollongong, Australia

**Keywords:** job characteristics, physical activity, well-being, tracking, wearables

## Abstract

The relation between job characteristics and health is one of the most important fields of research within work and organizational psychology. Another prominent variable influencing health is physical activity. The physical activity mediated Demand-Control (pamDC) model (Häusser & Mojzisch, 2017, https://doi.org/10.1080/02678373.2017.1303759) combines these health indicators in a new theoretical framework. Based on the pamDC model the current study aims to clarify the role of leisure time physical activity (LTPA) in the interplay of job demands, job control and well-being. We expect physical activity to partially mediate the impact of job characteristics on health. To avoid self-report bias considering physical activity we used a consumer fitness tracker to collect additional data. In total, 104 white-collar workers participated in the study. The results show that job control and job demands could predict well-being in cross-sectional analyses. In longitudinal analyses, this was only the case for job demands. Regarding the proposed mediating effect of LTPA between job characteristics and health, we could not detect a significant mediation in our sample. This was true for both self-reported and objective data on physical activity. This study provides a first step in validating the pamDC model and has implications for future research.

*Ex nihilo nihil fit* (“nothing can be created out of nothing”) is certainly valid. Applied in the context of work environment, it means that a company owes its productivity and success to the hard and dedicated work of motivated and healthy employees. Therefore, employers have good reasons to look after their employees. In 2017, employees in Austria were on sick leave for 12.5 days on average, with musculo-skeletal and respiratory diseases explaining 50% of all cases of sick leave (note that this average score does not include short-term sick leave of 1‒3 days; Leoni & Schwinger, 2017).

## The Job-Demand-Control (JDC) Model

There are multiple theories on how the workplace affects employees’ health, but one of the most significant models in the history of work and organizational psychology linking job characteristics to outcome variables such as stress, health, and well-being is the Job-Demand-Control Model (JDC model) from Karasek (1979). Job demands are referred to as workload or task pressure accentuating the quantitative demands and physical or emotional conditions accentuating the qualitative demands (Karasek, 1979; Karasek et al., 1998). Job control is defined as “the discretion permitted the worker in deciding how to meet these demands” (Karasek, 1979, p. 285). Karasek combines job demands and job control with two intensities each, high and low, to define four different types of jobs: 1) passive jobs—low demands and low control, 2) active jobs—high demands and high control, 3) low-strain jobs—low demands and high control, and 4) high-strain jobs—high demands and low control. The fourth type, high-strain jobs, have the most impact on physical and mental health. Several studies show that high-strain jobs increase the probability of developing psychosomatic symptoms and are associated with cardiovascular disease, poor physical health, poor vitality, poor social functioning, smoking, high fat intake, and poor states of mental health and well-being (e.g., Fila et al., 2017; Häusser et al., 2010; Hellerstedt & Jeffery, 1997; Karasek, 1979; Karasek et al., 1981; Lerner et al., 1994; Luchman & González-Morales, 2013; Morita & Wada, 2007).

## The Physical Activity Mediated Demand-Control Model (pamDC Model)

**Figure 1:**
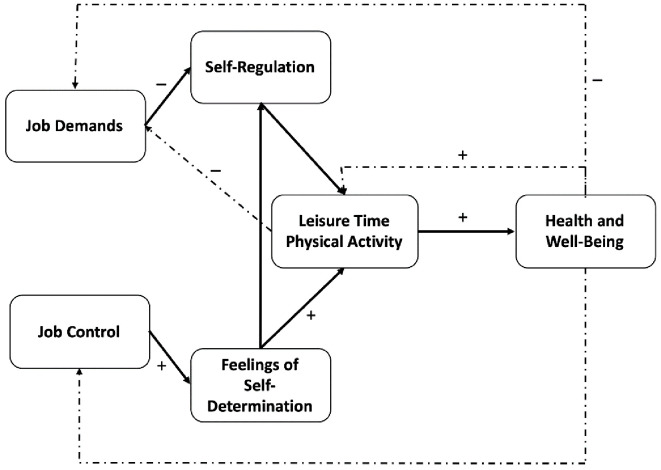
The Physical Activity Mediated Demand-Control (pamDC) Model (Häusser & Mojzisch, 2017)

Karasek’s JDC model focuses on the combination of job demands and job control to determine the impact of job strain on health and well-being, but other aspects might have an influence. This is why Häusser and Mojzisch (2017) developed a new theoretical framework which is originally based on the JDC model but also includes other variables e.g., leisure time physical activity (LTPA), self-regulation and feelings of self-determination. The physical activity mediated Demand-Control (pamDC) model is dedicated to filling a gap in research as “predicting well-being from job characteristics versus predicting well-being from LTPA have not been connected systematically despite their mutual aim to identify and describe antecedents of reduced well-being and impaired health” (Häusser & Mojzisch, 2017, p. 2). The goal of the pamDC model (see Figure 1) is to combine research on the effects of job characteristics on well-being and research on the effects of LTPA on well-being. To do so Häusser and Mojzisch (2017) recommend to examine the impact of job demands and job control on health and well-being separately. Furthermore, they expect a mediating role of physical activity within this relation. Based on findings in the literature Häusser and Mojzisch (2017) present eight propositions. These are aimed to be tested within the pamDC model and are presented here in short. For a detailed derivation see Häusser and Mojzisch (2017). Based on findings from Kirk and Rhodes (2012) and Popham and Mitchell (2007) where long working hours had a negative effect on physical activity, the pamDC model assumes that high job demands are associated with less LTPA (Proposition 1). They argue that “Dealing with high job demands typically requires self-regulation (e.g., remaining friendly, inhibiting temptations, remaining focused)” (Häusser & Mojzisch, 2017, p. 7) and therefore assume that a lack of self-regulatory fatigue mediates the effect of job demands on LTPA (Proposition 2). Considering job control, the authors propose based on empirical evidence (e.g., Bennett et al., 2006; Choi et al., 2010; Fransson et al., 2012) that job control is positively associated with LTPA (Proposition 3). Proposition 4 on the mediating effect of self-determination on the interplay of job control and LTPA is based on the self-determination theory (SDT), as autonomy is a key element in both self-determination and job control (Deci & Ryan, 1985). In their review of recent research on the JDC(-Support)-Model, Häusser et al. (2010) discuss the difference between main and interaction effects of job demands and job control and propose a buffering effect of job control on the negative effect of job demands on LTPA (Proposition 5). Following the findings of Moller et al. (2006) and Muraven et al. (2008) on self-determination and self-regulation, Häusser and Mojzisch (2017) expect an interaction of job control and self-regulatory fatigue when job demands are high (Proposition 6). With respect to empirical evidence on the interaction of LTPA and health, the authors expect a positive effect of LTPA on health and well-being (e.g., Lee et al., 2012; Lim et al., 2012; Moore et al., 2012; Pate et al., 1995), Proposition 7. Out of Propositions 1, 3, and 7, Häusser and Mojzisch (2017) expect a mediating effect of LTPA between job demands, job control and health and well-being (Proposition 8). In sum, the “pamDC model describes the relationships between workplace characteristics, LTPA, health, and well-being in terms of a causal chain: job demands and job control are predicted to affect LTPA (independently and interactively), and LTPA, in turn, is predicted to affect health and well-being” (Häusser & Mojzisch, 2017, p. 12). The authors nevertheless expect some reciprocal effects, as health-related problems could affect LTPA, or a high amount of LTPA could help people to efficiently cope with job demands, and consequently people might perceive job demands in a different way. Taking all these assumptions into account, the authors give some clear implications for future research regarding the pamDC model. As one core future implication, the authors state that it is not enough to consider only subjective information about physical activity, as research shows social desirability biases in recalling information (e.g., Canning et al., 2014; Donaldson & Grant-Vallone, 2002). To counteract these effects, the authors propose to additionally include measurement tools such as fitness trackers or accelerometers to collect objective data on LTPA in addition to subjective data on LTPA (Häusser & Mojzisch, 2017).

## Job Characteristics, Physical Activity, and Well-Being

Whereas job demands are found to be negatively associated with various health indicators, the opposite is true for physical activity. Research on cardiovascular disease has shown that a healthy lifestyle including physical activity on a regular basis reduces the risk of coronary artery disease (Eyler et al., 2003). The World Health Organization (WHO; WHO, 2006) recommends at least 30 minutes of moderate physical activity on a minimum of 5 days per week to maintain the status of health and well-being. In line with this, Pate et al. (1995) recommend 30 minutes of moderate physical activity every day of the week. Biddle (1995) concludes in a review on physical activity that it helps to reduce anxiety and depression and has a positive effect on self-esteem, self-concept, mood, and well-being. This conclusion is in line with the findings of Fox (1999), who also found that physical activity was positively associated with well-being in a clinical study on anxiety and depression. Wiese et al. (2018) accentuate in their meta-analysis the importance of active leisure time activities such as physical activity instead of passive activities (e.g., watching television). They found that LTPA was positively associated with subjective well-being. The authors argue that one possible reason might be the effort-recovery model (Meijman & Mulder, 1998), which proposes that recovery occurs when leisure activities need different resources from the work-related tasks, and due to digitalization, more people engage in sedentary jobs (e.g., Church et al., 2011; Wiese et al., 2018). In addition, Bakker, Demerouti et al. (2013) found an overall positive relationship between the total amount of LTPA and well-being in the evening. Research on job characteristics and LTPA shows that a high-demand work environment (high job demands and low job control) is associated with less LTPA (Kouvonen et al., 2005). Stults-Kolehmainen and Sinha (2014) similarly concluded in their systematic review of the effects of stress on physical activity that high-strain jobs are associated with less LTPA and more sedentary activities in leisure time. In sum, research on LTPA shows that 1) it is crucial for various physiological health indicators, 2) it is positively associated with psychological indicators, and 3) it is negatively associated with job characteristics such as job-related strain.

## Subjective and Objective Measurement of Physical Activity

We will not give a complete review of challenges when it comes to subjective or questionnaire-based information, as there already exist extensive reviews on that topic (see Ainsworth et al., 1994; Gonyea, 2005; Razavi, 2001; Sallis & Saelens, 2000), but we will give an overview of the most relevant challenges. Following the four-stage model of Tourangeau et al. (2000) on survey response behavior, there are several cognitive processes involved when using questionnaires as a tool of measurement: 1) comprehension of the question, 2) retrieval of relevant information from memory, 3) integration of judgement, and 4) response process by mapping the response (Tourangeau et al., 2000). Thus, answering a simple question from a survey requires different complex underlying processes. Considering the integration of judgement, social desirability plays a significant role; the person answering the question must decide whether he or she wants to tell the truth in accordance to the judgement he or she makes considering their actual individual state compared to the socially desired state (Tourangeau et al., 2000). Social desirability is one process that affects response behavior in questionnaires, but there are other processes that have an impact such as cultural differences or bias in memory retrieval (Choi & Pak, 2005; Johnson et al., 1997). Taking these challenges into account, the necessity for an objective method of measurement becomes clear. Addressing this issue in the field of physical activity, new technologies such as accelerometers, fitness trackers, and smart watches (generic term: wearables) might provide useful information or even serve as a future substitute for subjective data on physical activity. The potential benefits of wearables lie in the objective, continuous, non-invasive, and automatic way of collecting a variety of data in everyday life.

## The Present Study

In accordance with the empirical evidence presented above, the aim of the current study was to examine the effects of job characteristics on well-being and the proposed mediating effect of LTPA. We therefore assume that job demands are negatively associated with well-being, whereas the opposite is true for job control. We follow the proposition of the pamDC model and test this assumption separately for job demands and job control. Furthermore, we assume, that LTPA mediates the impact of job demands, job control on health and well-being. Considering the challenges of social desirability and recall bias in subjective measures, we follow the recommendations of Häusser and Mojzisch (2017). Therefore, we include objective measures of LTPA, by using a consumer fitness tracker, to test the proposed mediating effects.

## Method

### Participants and Procedure

Data collection was carried out in cooperation with a public institution in Austria. The field study was implemented as a project of occupational health promotion by combining subjective and objective health-related data. Two online surveys (T1 and T2) on LimeSurvey, an online survey tool, collected subjective data about several aspects including job characteristics, well-being, and LTPA. A consumer fitness tracker (XIAOMI Mi Band 2) collected objective data about LTPA in the form of steps. Participants were informed about the content and purpose of the study with an official presentation prior to data collection. Beforehand, employees received a letter of information about the study as they had to register to participate so we could provide enough fitness trackers. At the end of the opening presentation, participants signed an informed consent document, received the web link for the first online survey (T1) and their fitness tracker. For the following 14 days, participants wore their individual fitness trackers, and when they returned it, they received the web link for the second and final online survey (T2). In total, 104 employees (white-collar workers) participated in the field study. It should be noted that only participants of a certain department were able to participate in the study, and participation was voluntary. The education level of the sample was rather high with 81 participants (77.9%) having a college or university degree. Table 1 displays more details of the sample.

**Table 1:** Socio-Demographic Details of Participants (n = 104)

Characteristic	Number of participants	Percentage
Gender		
Male	50	48.1
Female	47	45.2
No statement	7	6.7
Age group		
< 25	0	0
25-29 years old	8	7.7
30-34 years old	6	5.8
35-39 years old	14	13.5
40-44 years old	6	5.8
45-49 years old	11	10.6
50-54 years old	19	18.3
55-59 years old	22	21.2
60-64 years old	11	10.6
No statement	7	6.7
Working hours		
≤ 25 hours/week	11	10.6
26-35 hours/week	9	8.7
≥ 36 hours/week	71	68.3
No statement	13	12.5
Member sports club/fitness center		
Yes	42	40.4
No	54	51.9
No statement	8	7.7
Marital status		
Married/in a relationship	75	72.1
Not married/not in a relationship	19	18.3
No statement	10	9.6

### Measures

#### Job Characteristics

Job demands and job control were measured with a shortened German version of the Copenhagen Psychosocial Questionnaire (COPSOQ; Nübling et al., 2005) at T1. Altogether, job demands were assessed with 14 items (e.g., “Is your workload unevenly distributed so it piles up?”; “Is your work emotionally demanding?”) and job control with 19 items (e.g., “Do you have a large degree of influence concerning your work?”; “Does your work require you to take the initiative?”). Items were answered on a scale with 0 (*never*), 25 (*rarely*), 50 (*sometimes*), 75 (*often*), and 100 (*always*). A mean score was computed from the subscales for job demands and job control for further analyses. Reliability (Cronbach’s α) of the two mean scores was high (α = .87 for job demands, α = .88 for job control).

#### Well-Being

Well-being was measured using the German version of the WHO-5 Well-being Index (Brähler et al., 2007). The WHO-5 consists of five items to be rated on a 6-point scale ranging from 0 (*at no time*) to 5 (*at all times*). The questionnaire contains items such as “I have felt calm and relaxed” and “My daily life has been filled with things that interest me.” The instruction for answering the items was “Please respond to each item by marking one box per row regarding how you felt in the last two weeks”. Cronbach’s α was high at both times of measurement (α = .85 at T1, α =.85 at T2).

#### Subjective LTPA

An adapted version of the Physical Activity Questionnaire (PAQ) was used to collect information about subjective LTPA (sLTPA) of the participants (Wagner & Singer, 2003). The questionnaire consists of 10 items, each asking about different types of physical activity: 1) walking fast, 2) Nordic walking or hiking, 3) jogging, 4) cycling, 5) gymnastics, 6) aerobics, 7) tennis, 8) skiing, 9) fitness-center, and 10) other physical activities. In the original version of the PAQ, the instruction is “How many hours per week did you spend on … during the last month?” As the time of measurement within this study was shorter, the instruction was adapted to “How much time (in minutes) did you spend on … during the last two weeks?” Therefore, participants rated for each activity the amount of time spent with one specific activity during the last two week in minutes. For further analysis, a mean score of subjective LTPA in minutes per day for each participant was computed. Subjective LTPA (sLTPA) was also measured two times (T1 and T2).

#### Objective LTPA

A consumer fitness tracker (XIAOMI Mi Band 2) collected objective data on participants’ LTPA in the form of steps. The XIAOMI Mi Band 2 is a smart device that can measure physical activity, heartrate, and sleep. It has a built-in motion sensor (pedometer) to measure steps and a built-in LED sensor to measure heartrate. To ensure that the data collected with the fitness tracker would exclusively be available for the research team of this study and would not at any time be transferred elsewhere (e.g., to the XIAOMI company or a third-person server), the fitness trackers were put in operation exclusively with our own application. The basis of our own app was the open source app, Gadgetbridge for Android, which was installed on an Acer Iconia One 10 and a Samsung Galaxy Tab S3. The app allows using smart devices (e.g., the XIAOMI Mi Band 2) without creating a user account or using the closed-source app of the vendors. Aside from the crucial benefit of data protection when using Gadgetbridge as a basis of our app, the source code could be adjusted to fit the purpose of the study. As we wanted the data to be as natural as possible, we adjusted the fitness tracker settings via the app so that the steps would not show on the display of the fitness tracker but only the current time, battery level, and heartrate. During data collection, all data were saved and kept on the built-in memory chip of the fitness tracker. When participants returned their fitness tracker at the end of the study, we transferred the data onto the tablet and saved it on a protected server at the university. For statistical analyses, a mean score of objective LTPA (oLTPA) in steps per day for all participants was computed.

## Results

### Descriptive Data

Data management and analysis were performed using SPSS (Version 20.0). Table 2 illustrates means, standard deviations, sample sizes, and correlations of the predictor variables job demands, job control, subjective physical activity (sLTPA) in minutes (T1 and T2) and objective physical activity (oLTPA) in steps as well as the criterion variable well-being (T1 and T2). Well-being at both times showed the expected relation with job characteristics; job demands were negatively associated with well-being, whereas job control was positively associated with well-being. Therefore, it appears that participants with lower job demands or with higher job control felt better than the ones with higher job demands or lower job control. Furthermore, well-being at T1 was positively related to sLTPA at T1, suggesting that participants who were more active also felt better. Objective LTPA in the form of steps showed a significant but rather low correlation with sLTPA at T2.

**Table 2:** Descriptive Statistics and Correlations of Study Variables

Variable	*M*	*SD*	*n*	1.	2.	3.	4.	5.	6.
1. Job demands	37.35	15.92	98	-					
2. Job control	65.56	13.16	98	-.30**	-				
3. sLTPA T1	42.07	35.46	95	-.09	-.01	-			
4. sLTPA T2	50.41	50.50	97	-.05	-.10	.56**	-		
5. oLTPA T2	5545	3686	96	-.09	-.11	.09	.24*	-	
6. Well-being T1	2.83	0.96	98	-.49**	.49**	.24*	.15	.04	-
7. Well-being T2	3.06	0.91	99	-.49**	.39**	.19	.17	-.01	.74**

*Note*. sLTPA = subjective leisure time physical activity (minutes per day); oLTPA = objective leisure time physical activity (steps per day); T1 = first online survey; T2 = second and final online survey.

*p < .05. **p < .001.

### Regression Analyses

Multiple regression analysis was used to test if job demands and job control predicted participants’ well-being. The results of the multiple regression analyses on the prediction of well-being at T1 indicated that the two predictors explained 37.8% of the variance, *F*(2, 95) = 28.83, *p* < .001. It was found that job demands and job control significantly predicted well-being at T1 (see Table 3). The results of multiple regression analyses on the prediction of well-being at T2 showed that the predictors could explain 56.7% of the variance in well-being at T2, *F*(3, 89) = 38.88, *p* < .001. In detail the results show that job demands and well-being at T1 significantly predicted well-being at T2 (see Table 3).

**Table 3:** Results of Linear Regression Analyses for Job Demands and Job Control Predicting Well-Being at T1 and T2 (n = 93)

Outcome variable/Predictor	*B*	*SE B*	β	*t*	*p*
Well-being T1					
Job demands T1	-0.02	0.01	-.38	-4.54	< .001
Job control T1	0.03	0.01	.38	4.45	< .001
Well-being T2					
Job demands T1	-0.01	0.00	-.17	-2.15	.034
Job control T1	0.00	0.01	.05	0.57	.567
Well-being T1	0.60	0.08	.63	7.26	< .001

*Note*. T1 = first online survey; T2 = second and final online survey.

### Mediation Analyses

**Figure 2:**
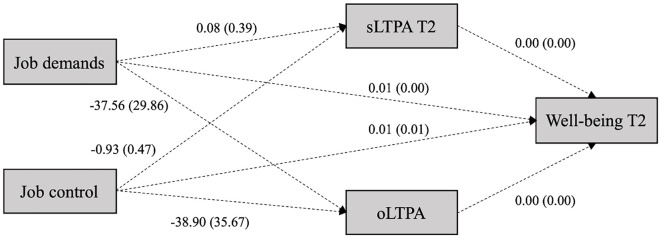
Unstandardized Regression Coefficients of Mediation Analysis of Job Demands and Job Control on Well-Being via Subjective and Objective LTPA *Note*. Standard errors in parenthesis (*n* = 86). sLTPA = subjective LTPA (minutes per day); oLTPA = objective LTPA (steps per day); T2 = second and final online survey.

Mediation analyses were performed using the PROCESS macro in SPSS to test if the relation between job demands, job control, and well-being was partially mediated by subjective and objective LTPA in longitudinal data (Hayes, 2012). The PROCESS macro uses bootstrapping to determine mediation effects (indirect effects). Confidence intervals and estimates were calculated over 5,000 bootstrapped resamples with a 95% confidence interval. The mediation analysis included job demands and job control as predictor variables, subjective and objective LTPA (sLTPA, oLTPA) as mediator variables, well-being at T1 as a control variable, and well-being at T2 as the criterion. Unstandardized regression coefficients did not achieve statistical significance (see Figure 2).

The results show that 64% of the variance in well-being at T2 could be predicted through the variables, *F*(5, 85) = 28.63, *p* < .001 (see Table 4). Mediation effects (indirect effects) did not achieve statistical significance, as the criterion was a 95% bootstrapped confidence interval excluding zero (see Table 4).

**Table 4:** Multiple Mediation Analysis of Job Demands and Job Control on Well-Being via Subjective and Objective LTPA (n = 86)

Predictor	Longitudinal			
	*B*	*SE*	*p*	95% CI
Control variables				
Well-being T1	0.627	0.079	< .001	
Job demands *c´*	-0.005	0.004	.262	
(total effect *c*)	-0.005	0.004	.309	
Job Control *c´*	0.006	0.005	.235	
(total effect *c*)	0.005	0.005	.319	
Mediator variables				
sLTPA T2	0.002	0.001	.179	
oLTPA T2	0.000	0.000	.536	
Indirect effects (job demands)				
sLTPA T2	0.000	0.001		[-0.001, 0.001]
oLTPA T2	0.000	0.001		[-0.000, 0.001]
Indirect effects (job control)				
sLTPA T2	-0.002	0.002		[-0.005, 0.001]
oLTPA T2	0.000	0.001		[-0.000, 0.002]
*R* ^2^	.638		< .001	

*Note.* sLTPA = subjective LTPA (minutes per day); oLTPA = objective LTPA (steps per day); T1 = first online survey; T2 = second and final online survey.

## Discussion

In line with findings in the literature, our study showed that job demands and job control contributed to the prediction of well-being in a cross-sectional analysis, explaining about 37.8% of the variance. Considering the longitudinal analysis of well-being, only job demands and well-being at T1 were significant predictor variables, explaining 57% of the variance. It must be mentioned that the sample of the current study was rather homogenous: All 104 participants were employed at the same department with working outside the office as the main component of work. Therefore, it seems likely that participants are used to high levels of decision latitude considering time structure and work procedure.

Another factor that might have contributed to an increase in well-being is that participants knew they were actively taking part in a study on workplace health promotion. This knowledge might have influenced participants’ behavior towards a healthier lifestyle resulting in better well-being at the end of the study. The effect that people change behavior when they know they are being observed is also known as the Hawthorne effect (Landsberger, 1958).

Mediation analyses showed that subjective and objective LTPA did not significantly mediate the effect of job demands or job control on well-being. This result fails to support the pamDC model of Häusser and Mojzisch (2017) in which a partial mediation of physical activity between job characteristics and well-being was proposed. There could be multiple reasons why data on physical activity could not predict well-being in the current study. As subjective physical activity at T1 and T2 were reported retrospectively for the past two weeks, the information could have been affected by recall bias. A recall bias can occur in surveys when information about a certain criterion is over- or under-reported, which can be intended or unintended (Basso et al., 1997; Swan et al., 1992). Especially when it comes to social desirability considering a healthy and sportive lifestyle, subjective survey information can be biased.

Another possible reason for our null findings might be that not all types of physical activity have the same impact on well-being. For instance, WHO recommends at least 2.5 hours of moderate-intensity physical activity, whereas the PAQ also includes low-intensity physical activity such as walking fast (Wagner & Singer, 2003; WHO, 2006). Another finding of the current study is the rather low but significant correlation of subjective physical activity and objective physical activity. One main reason for the rather low correlation of subjective and objective physical activity might be that not all types of sports are recognized by the measure of steps on a fitness tracker. The sensors in the fitness tracker measure motion in a specific way, deciding whether a certain motion is considered a step or not. It is worn on the wrist, so in sports with less movement of the upper body, the fitness tracker might not count this physical activity as steps. This is especially true for bicycling but also for sports such as Yoga or Pilates. The definition of physical activity in the pamDC model is a broader one including walking instead of using a car and the steps taken during working hours to physical activity (Häusser & Mojzisch, 2017). When asking participants about their subjective physical activity, it might be useful to do so on a daily basis in order to get more detailed information for comparison with objective physical activity from the fitness tracker.

Regarding the use of self-tracking devices to measure physical activity rather than using self-report measures, the current study shows empirically that steps are insufficient to substitute for questionnaires on physical activity. However, subjective and objective measures show at least a significant correlation, indicating that steps counted during leisure time are at least partly an indication for an active lifestyle. The shortcomings of steps not including specific sports and activities (e.g., swimming, cycling) while including non-sportive activities (e.g., brushing your teeth, applauding) suggest that self-report questionnaires yet cannot be completely replaced by a consumer fitness tracker for research purposes. There is a need for devices that manage to measure and differentiate between specific kinds of movement, whether the current movement is part of a sportive or a non-sportive activity. In a context in which LTPA is measured, non-sportive activity is not necessarily as relevant to the study as sportive activity and should be acknowledged as such. In circumstances where only a trend in increased or decreased physical activity is of interest, a pedometer could possibly be sufficient, but many activities remain unmeasured. Furthermore, it should be clearly acknowledged that even with the inaccuracy reported, steps taken over a period of time are indicators of some form of physical movement. Accumulated steps over a period of time might be of interest when thinking of general physical activity as opposed to specific sportive activities.

Recognizing the limitations of the current study, the results provide a first step toward validating the pamDC model (Häusser & Mojzisch, 2017) and integrating objective measures of physical activity in the process.
